# Implementation research on community health workers’ provision of maternal and child health services in rural Liberia

**DOI:** 10.2471/BLT.16.175513

**Published:** 2017-02-01

**Authors:** Peter W Luckow, Avi Kenny, Emily White, Madeleine Ballard, Lorenzo Dorr, Kirby Erlandson, Benjamin Grant, Alice Johnson, Breanna Lorenzen, Subarna Mukherjee, E John Ly, Abigail McDaniel, Netus Nowine, Vidiya Sathananthan, Gerald A Sechler, John D Kraemer, Mark J Siedner, Rajesh Panjabi

**Affiliations:** a Geisel School of Medicine at Dartmouth College, Hanover, United States of America (USA).; b Last Mile Health, Monrovia, Liberia.; c Department of Social Policy and Intervention, University of Oxford, Oxford, England.; d Harvard Medical School, Boston, USA.; e University of Minnesota Medical School, Minneapolis, USA.; f Grand Gedeh County Health Team, Ministry of Health, Monrovia, Liberia.; g Division of Global Health Equity, Brigham and Women’s Hospital, Harvard Medical School, 75 Francis Street, Boston, MA, 02115, USA.; h Department of Health Systems Administration, Georgetown School of Nursing and Health Studies, Washington, USA.; i Department of Medicine, Harvard Medical School, Boston, USA.

## Abstract

**Objective:**

To assess changes in the use of essential maternal and child health services in Konobo, Liberia, after implementation of an enhanced community health worker (CHW) programme.

**Methods:**

The Liberian Ministry of Health partnered with Last Mile Health, a nongovernmental organization, to implement a pilot CHW programme with enhanced recruitment, training, supervision and compensation. To assess changes in maternal and child health-care use, we conducted repeated cross-sectional cluster surveys before (2012) and after (2015) programme implementation.

**Findings:**

Between 2012 and 2015, 54 CHWs, seven peer supervisors and three clinical supervisors were trained to serve a population of 12 127 people in 44 communities. The regression-adjusted percentage of children receiving care from formal care providers increased by 60.1 (95% confidence interval, CI: 51.6 to 68.7) percentage points for diarrhoea, by 30.6 (95% CI: 20.5 to 40.7) for fever and by 51.2 (95% CI: 37.9 to 64.5) for acute respiratory infection. Facility-based delivery increased by 28.2 points (95% CI: 20.3 to 36.1). Facility-based delivery and formal sector care for acute respiratory infection and diarrhoea increased more in agricultural than gold-mining communities. Receipt of one-or-more antenatal care sessions at a health facility and postnatal care within 24 hours of delivery did not change significantly.

**Conclusion:**

We identified significant increases in uptake of child and maternal health-care services from formal providers during the pilot CHW programme in remote rural Liberia. Clinic-based services, such as postnatal care, and services in specific settings, such as mining areas, require additional interventions to achieve optimal outcomes.

## Résumé

**Objectif:**

Évaluer les changements dans le recours aux services essentiels de santé maternelle et infantile à Konobo, au Libéria, après la mise en œuvre d'un programme de perfectionnement des agents de santé communautaires.

**Méthodes:**

Le ministère de la Santé libérien s'est associé avec Last Mile Health, une organisation non gouvernementale, afin de mettre en œuvre un programme pilote destiné à améliorer le recrutement, la formation, l'encadrement et la rémunération des agents de santé communautaires. Pour évaluer les changements au niveau des soins de santé maternelle et infantile, nous avons réalisé plusieurs sondages transversaux par grappes avant (2012) et après (2015) la mise en œuvre du programme.

**Résultats:**

Entre 2012 et 2015, 54 agents de santé communautaires, sept collègues superviseurs et trois superviseurs cliniques ont été formés pour prendre en charge une population de 12 127 personnes dans 44 communautés. Le pourcentage corrigé par régression d'enfants recevant des soins de la part de prestataires de soins officiels a augmenté de 60,1 (intervalle de confiance (IC) de 95%: 51,6 à 68,7) points de pourcentage pour la diarrhée, de 30,6 (IC 95%: 20,5 à 40,7) pour la fièvre et de 51,2 (IC 95%: 37,9 à 64,5) pour les infections aiguës des voies respiratoires. Les accouchements en maternité ont augmenté de 28,2 points (IC 95%: 20,3 à 36,1). Les accouchements en maternité et la prise en charge dans le secteur formel des infections aiguës des voies respiratoires et des cas de diarrhée ont davantage augmenté dans les communautés agricoles que dans celles vivant de l'extraction de l'or. Nous n’avons pas observé de changements significatifs dans le fait de bénéficier d'une ou plusieurs séances de soins prénataux dans une structure de soins ou de soins post-partum dans les 24 heures suivant l'accouchement.

**Conclusion:**

Nous avons observé une augmentation significative de la prestation de services de santé maternelle et infantile par des prestataires officiels lors du programme pilote destiné aux agents de santé communautaires dans les régions rurales isolées du Libéria. Des interventions supplémentaires sont nécessaires pour obtenir des résultats optimaux vis-à-vis de la prestation de services cliniques, comme les soins post-partum, et de services dans des zones spécifiques, comme les régions minières.

## Resumen

**Objetivo:**

Evaluar los cambios en el uso de servicios de salud materna e infantil básicos en Konobo, Liberia, tras la implementación de un programa mejorado de trabajadores comunitarios de salud (CHW, por sus siglas en inglés).

**Métodos:**

El Ministerio de Salud de Liberia se asoció con *Last Mile Health*, una organización no gubernamental, para implementar un programa piloto de CHW con una mejora en la contratación, formación, supervisión y compensación. Para evaluar los cambios en el uso de la atención sanitaria materna e infantil, se realizaron repetidas encuestas transversales de conglomerados antes (2012) y después (2015) de la implementación del programa.

**Resultados:**

Entre 2012 y 2015, 54 CHW, 7 supervisores homólogos y 3 supervisores clínicos recibieron formación para trabajar para una población de 12 127 personas en 44 comunidades. El porcentaje ajustado a la regresión de niños que recibieron atención de profesionales sanitarios formales aumentó un 60,1% (intervalo de confianza (IC) del 95%: 51,6 a 68,7) para la diarrea, un 30,6% (IC del 95%: 20,5 a 40,7) para la fiebre y un 51,2% (IC del 95%: 37,9 a 64,5) para la infección respiratoria aguda. El suministro en centros aumentó un 28,2% (IC del 95%: 20,3 a 36,1). El suministro en centros y la atención del sector formal para la infección respiratoria aguda y la diarrea aumentaron más en comunidades agrícolas que en las de minas de oro. La recepción de una o más sesiones de atención prenatal en un centro sanitario y de atención posparto a las 24 horas del parto no experimentó cambios importantes.

**Conclusión:**

Se identificaron aumentos significativos en la aceptación de los servicios de atención sanitaria materna e infantil de profesionales formales durante la versión piloto del programa de CHW en la Liberia rural remota. Los servicios clínicos, como la atención posparto y los servicios en lugares concretos, como zonas mineras, requieren intervenciones adicionales para lograr unos resultados óptimos.

## ملخص

**الغرض:**

تقييم التغييرات التي طرأت على الاستعانة بالخدمات الضرورية لصحة الأمهات والأطفال في كونوبو بليبيريا بعد تنفيذ برنامج محسّن للأخصائيين الصحيين للمجتمع المحلي.

**الطريقة:**

دخلت وزارة الصحة الليبيرية في إطار من الشراكة مع منظمة "لاست مايل هيلث" الصحية غير الحكومية لتنفيذ برنامج تجريبي للأخصائيين الصحيين للمجتمع المحلي مع تطوير عمليات التعيين والتدريب والإشراف وتقديم الأجور. ولكي يتم تقييم التغييرات في مجال الاستعانة بالرعاية الصحية للأمهات والأطفال، فقد أجريت مجموعة من الاستبيانات المجمعة القطاعية المتكررة قبل تنفيذ البرنامج (في عام 2012) وبعد تنفيذه (في عام 2015).

**النتائج:**

في الفترة ما بين عاميّ 2012 و2015 تم تدريب 54 من الأخصائيين الصحيين للمجتمع المحلي، وسبعة مشرفين من النظراء، وثلاثة مشرفين سريريين لتقديم الخدمة لشريحة سكانية يبلغ قوامها 12،127 نسمة في 44 مجتمعًا محليًا. وقد زادت النسبة المئوية المعدّلة وفقًا لنموذج الانحدار الإحصائي للأطفال الذين يتلقون الرعاية من مقدمي الرعاية الرسميين بمقدار 60.1 (بمستوى ثقة تبلغ نسبته 95‏%: 51.6 إلى 68.7) نقطة مئوية بالنسبة للإسهال، وبمقدار 30.6 (بمستوى ثقة تبلغ نسبته ‏95%: 20.5 إلى 40.7) نقطة مئوية للحمى، وبمقدار 51.2 (بمستوى ثقة تبلغ نسبته ‏95%: 37.9 إلى 64.5) للعدوى التنفسية الحادة. كما تزايد إجراء عمليات الولادة في المرافق الصحية بمقدار 28.2 نقطة (بمستوى ثقة تبلغ نسبته 95‏%: 20.3 إلى 36.1). وقد تحققت زيادة أكبر في نسبة عمليات الولادة في المرافق الصحية والرعاية المقدمة من جانب الجهات الرسمية لحالات العدوى الحادة في الجهاز التنفسي والإسهال في المجتمعات المحلية الزراعية بالمقارنة مع المجتمعات المحلية القائمة على تعدين الذهب. ولم تطرأ زيادة ملموسة على تلقي جلسات الرعاية لمرحلة ما قبل الولادة بمعدل جلسة واحدة أو أكثر في المرافق الصحية وخدمات الرعاية بعد الولادة خلال فترة 24 ساعة من الولادة.

**الاستنتاج:**

لقد حددنا زيادات ملموسة في حجم تلقي خدمات الرعاية الصحية للأمهات والأطفال من الجهات الرسمية خلال فترة البرنامج التجريبي للأخصائيين الصحيين للمجتمع المحلي في المناطق الريفية النائية في ليبيريا. وتحتاج الخدمات المعتمدة على العيادات – مثل الرعاية في مرحلة ما بعد الولادة - والخدمات المقدمة في بيئات محددة، مثل مناطق التعدين إلى تدخلات إضافية لتحقيق أفضل النتائج.

## 摘要

**目的:**

旨在评估实施加强的社区卫生工作者 (CHW) 计划以后，利比里亚科诺博地区基本妇幼卫生服务使用情况的变化。

**方法:**

利比里亚卫生部联手最后一里医疗 (Last Mile Health)，一家非政府组织，实施在招募、培训、监督和报酬方面均有所加强的社区卫生工作者 (CHW) 试点计划。 为了评估妇幼保健使用的变化，我们在该计划实施之前 (2012) 和之后 (2015) 分别开展了重复性横断面群体调查。

**结果:**

2012 至 2015 年间，54 名社区卫生工作者、七名同伴监管员和三名临床监管员接受了培训，以服务 44 个社区内的 12 127 名居民。 从正规护理提供者处接受护理的腹泻儿童的回归调整百分比提高了 60.1【95% 置信区间 (CI)： 51.6 至 68.7】个百分点，发热儿童百分比提高了 30.6（95% CI： 20.5 至 40.7），急性呼吸道感染儿童的百分比提高了 51.2 （95% CI： 37.9 至 64.5）。 入院分娩提高了 28.2 个百分点（95% CI： 20.3 至 36.1）。 与采金地区相比，农业地区入院分娩和前往正规科室治疗急性呼吸道感染和腹泻的情况增加得更多。 在医疗机构接受一项或多项产前护理课程和产后 24 小时内护理的情况没有显著变化。

**结论:**

我们发现利比里亚偏远农村地区在社区卫生工作者 (CHW) 计划试点期间从正规医疗机构接受妇幼保健服务的情况显著增加。 腹泻护理等临床服务和采矿地区等特定环境下的服务，需要额外的干预才能达到最理想的效果。

## Резюме

**Цель:**

Оценить изменения в использовании основных услуг в сфере охраны здоровья матерей и детей в округе Конобо, Либерия, после внедрения усовершенствованной программы для местных медицинских работников (ММР).

**Методы:**

Министерство здравоохранения Либерии вступило в партнерские отношения с неправительственной организацией Last Mile Health для внедрения пробной программы ММР, предполагающей усовершенствования в наборе кадров, обучении, надзоре и оплате труда. Чтобы оценить изменения в использовании услуг в сфере охраны здоровья матерей и детей, авторы провели несколько межсекторальных обследований с применением гнездовой выборки до (2012 год) и после (2015 год) внедрения программы.

**Результаты:**

В период между 2012 и 2015 годами было подготовлено 54 ММР, семь инспекторов из партнерских организаций и три инспектора из клиник для обслуживания 12 127 человек в 44 сообществах. Скорректированная, рассчитанная с помощью модели регрессии доля детей, которые получили медицинскую помощь от официальных работников здравоохранения, увеличилась на 60,1 (95% доверительный интервал, ДИ: 51,6–68,7) процентного пункта в случае с диареей, на 30,6 (95% ДИ: 20,5–40,7) при лихорадке и на 51,2 (95% ДИ: 37,9–64,5) при острых респираторных инфекциях. Доля родов, принятых в учреждениях, увеличилась на 28,2 пункта (95% ДИ: 20,3–36,1). Доля родов, принятых в учреждениях, и помощи, оказанной со стороны официального сектора при острых респираторных заболеваниях и диарее, увеличилась больше в сельскохозяйственных общинах, чем в золотодобывающих. Изменения в доле получивших дородовую медицинскую помощь в рамках одного или нескольких сеансов в медицинском учреждении и послеродовой уход в течение 24 часов после родов не были статистически значимы.

**Вывод:**

Авторы выявили существенное увеличение в использовании услуг в сфере охраны здоровья матерей и детей, оказываемых официальными медицинскими работниками, в ходе реализации пробной программы ММР в удаленных сельских регионах Либерии. Клинические услуги, такие как послеродовой уход, и обслуживание в специфических условиях, таких как районы разработки полезных ископаемых, требуют осуществления дополнительных вмешательств для достижения оптимального результата.

## Introduction

Over 95% of global maternal and child deaths occur in 75 low- and middle-income countries and remote populations within these countries often bear the greatest burden.^1^^,^^2^ Many countries are exploring strategies to scale up community health worker (CHW)-based programmes, which have been demonstrated to improve health in the domains of maternal and child health, access to family planning and prevention of human immunodeficiency virus (HIV) infection, malaria and tuberculosis.^3^^,^^4^

In Liberia, an estimated 60% (1.2 million people) of the rural population lives more than 5 km from the nearest health facility and the country has among the highest maternal and child mortality rates globally, 725 deaths per 100 000 live births and 70 deaths per 1000 live births, respectively.^5^^–^^7^ In 2012, the health ministry partnered with Last Mile Health, a nongovernmental organization, to pilot a programme for enhanced CHW-based health care for remote populations (those living farther than 5 km or a one hour walk from the nearest health facility). The programme aimed to increase coverage of essential maternal and child health services through enhanced recruitment, training, supervision and compensation of CHWs. Responsibilities of CHWs included provision of: (i) integrated community case management of childhood illnesses, including diarrhoea, acute respiratory infection and malaria; and (ii) maternal and newborn care. Here, we describe the programmatic components, implementation and an assessment of changes in maternal and child health-care use three years after implementation.

## Methods

### Setting and participants

The programme took place in Konobo district in south-eastern Liberia. Konobo is one of Liberia’s most remote regions, comprised of 2983 km^2^ of rainforest, with a population density of 4.1 people/km^2^. In 2012, approximately 12 000 residents in the district lived more than 5 km from the nearest clinic. One quarter were women of reproductive age (15–49 years) and 16% were children under five years. These two demographic groups represented the target population of the programme. All 44 remote communities in Konobo, located more than 5 km from the district’s only health clinic were involved with the programme. The average road distance from these communities to the clinic was approximately 25 km and the mean population of the communities was 276 people. According to the 2012 Liberian Demographic and Health Survey, Konobo had worse maternal and child health outcomes than other rural Liberian districts.^8^

### Implementation

We implemented the programme between 2012 and 2015 in a stepwise fashion over three geographic areas within Konobo district. Integrated community case management of childhood illness was launched in February 2013, August 2013 and March 2014. Maternal and newborn care services were launched in November 2012, December 2013 and April 2015.

### CHW recruitment and staffing

We recruited CHWs through community nomination, as recommended by the 2008 *National policy and strategy on community health services*.^9^ Our recruitment process added several components, including (i) completion of a literacy test; (ii) an in-person interview to assess motivation and communication skills; and (iii) training and subsequent skills’ assessment for candidates that passed the interview. After this additional three-step screening and assessment, we identified and hired the highest scoring CHWs. We also conducted a follow-up competency assessment during the first 90-days of their employment. We recruited CHWs from the communities in which they resided, and they served communities within a 30-minute walk from their home community.

We also interviewed and hired two types of supervisors: clinical supervisors (i.e. clinic-based community health nurses and physician assistants) and peer supervisors (i.e. non-clinical supervisors who conduct process supervision and community engagement). Best performing CHWs were promoted to serve as peer supervisors.

### Training, supervision and compensation

CHWs completed an initial two-week training in the district capital. Training modules focused on community leadership (to promote community engagement), household mapping (to define his/her catchment population) and registration (to assess demographics). Subsequent modules were administered in the district capital roughly once every three months and focused on preventive and curative components of maternal, neonatal and child health services, including birth planning, perinatal care, integrated community case management of malaria, acute respiratory infection and diarrhoea, and criteria for referral of patients with warning signs to health clinics. A typical training took two weeks to complete. Physician assistants and registered nurses led the training sessions that focused on clinical skills, such as history-taking, physical examination and specific clinical procedures, such as rapid diagnostic testing for malaria. After the start of the Ebola virus disease outbreak, we added a training module on surveillance for Ebola symptoms.

CHWs received weekly supervision visits from peer supervisors who were trained in project and supply management, supportive supervision and referrals. Supervision visits were designed to last one hour each and consisted of form reviews, patient audits and restocking of essential commodities. High-performing CHWs were promoted to peer supervisors, and were equipped with a motorbike for travel during supervision visits. Initially, the visits were unstructured and left to the discretion of individual supervisors, however, since May 2013, supervision visits included the use of quality assurance checklists and randomly-sampled patient audits led by the peer supervisors. Separately, clinical supervisors conducted monthly field supervision visits to assess adherence to clinical protocols and provide formative feedback based on form reviews and direct observation of patient interactions.

Although the 2008 *National policy and strategy on community health services* specified in-kind compensation for CHWs,^9^ an agreement with the health ministry allowed an additional monthly cash payment to CHWs and supervisors. CHWs were paid 60 United States dollars (US$) per month for an estimated 20 hours of work per week, while peer supervisors and clinical supervisors were paid US$ 150 and US$ 550 per month respectively.

### Programme services

Initially, services were provided through passive surveillance, whereby community members visited CHW households during periods of illness or pregnancy. CHWs were trained to conduct integrated community case management for diarrhoea, acute respiratory infection and malaria, along with referral of cases that presented with danger signs. They were equipped with diagnostic tools, including rapid malaria diagnostic tests, mid-upper arm circumference bands and thermometers, and therapeutics, including zinc, oral rehydration salts, amoxicillin, acetaminophen and artemisinin-based combination therapy. Malaria was diagnosed with rapid diagnostic tests in children, with a measured fever. Additionally, CHWs conducted home-based antenatal care education, helped design birth plans, scheduled facility-based deliveries, screened pregnant women and neonates for danger signs, referred cases with danger signs to the clinic and promoted exclusive breastfeeding. CHWs provided services at no cost to community members. To further promote health-care utilization, CHWs organized community health committees that partnered with trained traditional midwives to refer expectant mothers to stay at maternal waiting homes (a residence near the clinic for at-term mothers), until childbirth. Beginning in April 2013, the programme paid the midwives US$ 3–5 for clinic referrals, and mothers who delivered in the clinic were provided transport reimbursements and food stipends.

We modified several elements of the programme during the Ebola virus disease outbreak. After the start of the outbreak in 2015, CHWs performed active surveillance through monthly household visits. While there were no confirmed cases of Ebola in the study area, use of rapid malaria diagnostic tests was suspended to ensure the safety of CHWs, and the programme adopted treatment protocols based on self-reported signs and symptoms. Similar changes were implemented for treatment of diarrhoea and acute respiratory infection.

### Data collection and analysis

We used data from two population-representative household surveys conducted by Last Mile Health in August 2012 and August 2015. Fundamental aspects of the survey design and execution were described previously.^2^ The questionnaire was adapted from the 2007 and 2012 Liberian Demographic and Health Surveys and included sections on household characteristics, maternal and neonatal health, reproductive health, child health and access to health care. We used a two-stage cluster design for the sampling, which provided a representative sample for assessing changes in maternal and child health-care use. We constructed a sampling frame using raw data from the 2008 Liberian Census. The frame was adjusted using information from household enumeration performed by Last Mile Health before each survey. Communities were the primary sampling units and were selected using probability-proportional-to-size sampling. Individual households served as secondary sampling units. Random selection of households within communities was done through a random walk procedure. For the 2012 baseline survey, we had a total sample of 600 households, selected from 30 clusters. Last Mile Health updated the sampling frame for the 2015 follow-up survey after a re-count of all the households in the district. The 2015 sample included 1035 households, selected from 45 clusters. We interviewed women ages 18–49 years in both surveys.

We made certain changes to the survey between 2012 and 2015. We added questions to the follow-up survey on asset ownership, family planning, provider use, vaccination and knowledge of Ebola. Before and after implementation, comparisons were restricted to consistent items between surveys and to communities common to both sampling frames. Individual weights for survey variables were adjusted post-hoc based on 2015 data. In 2012, enumerators interviewed the woman in the household who most recently completed a pregnancy, while in 2015, all women within a household were sampled and interviewed. The comparative analysis was therefore restricted to the household woman who in the 2015 survey responded as giving birth most recently. Surveys were done in Liberian English and Konobo Krahn by bilingual enumerators.

### Outcome measures

We defined the child health-care use outcomes as management of childhood illnesses by a formal care provider within a two-week recall period. The childhood illnesses included were: (i) diarrhoea; (ii) acute respiratory infection (defined as the combination of fever with a rapid respiratory rate); and (iii) fever. We defined formal care providers as community health workers, ministry of health community health volunteers (who were active in some parts of Liberia but not in our study area) and clinic staff. For maternal and neonatal health-care use, we defined outcomes as: (i) completing at least one antenatal care visit at a health facility; (ii) having a facility-based delivery; and (iii) receiving postnatal care from a clinic staff member or a CHW within 24 hours of delivery. To assess change in child health-care use, data from the 2012 survey and the 2015 survey were used as before and after programme implementation, respectively. To assess changes with maternal care use, the 2012 survey was used as a baseline, but assessment of programme implementation was restricted to births captured after April 2013, when all catchment communities had initiated at least one maternal health programme element.

### Data analysis

We conducted descriptive analyses to summarize respondent characteristics at baseline and after programme implementation. We fit logistic regression models to compare differences in each of the outcome indicators before and after implementation. The regression models for maternal health were adjusted for community type (agricultural versus gold-mining), maternal age, distance to health facility (measured by global positioning system) and presence or absence of motor vehicle access to the nearest health facility. The models for child health were adjusted for these same variables as well as the child’s age. After regression, we used predictive margins, holding covariates at their observed values to estimate adjusted percentages of each outcome indicator before and after programme implementation, and tested before-to-after changes using contrasts of predicted percentages. Since the CHW programme had not started at the time of the baseline survey in 2012, we estimated the percentage of child health encounters for integrated community case management of childhood illnesses that were provided by a CHW only for 2015. To assess moderating effects of community type, we ran the same maternal and child health models with an interaction term of community type and programme period. All analyses incorporated complex sampling design using inverse probability weights and finite population corrections at both stages. Standard errors were adjusted for clustering using Taylor linearization. Statistical analyses were conducted using Stata version 14.2 (Statacorp, College Station, Texas, United States of America).

### Ethical considerations

We obtained ethical approval for the surveys from the institutional review boards of Partners Healthcare, Georgetown University and the Liberian Institute for Biomedical Research. Respondents gave verbal informed consent.

## Results

### Implementation of programme

Between October 2012 and August 2015, we recruited and trained a total of 54 CHWs and 10 supervisors, of whom 39 CHWs and 5 supervisors remained active at the end of the pilot period. By the completion of the programme, CHW-to-population ratio within the study area was 1:311, which was more than threefold higher than the ratio of 1:1 000 proposed in the Liberian health ministry’s policy.^9^

### Respondent characteristics

Table 1 summarizes the survey respondents’ characteristics. We used data from 364 women of the 2012 survey and 205 women, who met the inclusion criteria of the 2015 survey. The mean maternal ages were 30 and 29 years (*P* = 0.002), respectively. Completing secondary education was more common in the follow-up survey (*P* = 0.039). There were no statistically significant differences in other demographic characteristics. The number of children who met the inclusion criteria was 470 in 2012 and 452 in 2015.

**Table 1:** Demographic characteristics of respondents and description of the survey site, by survey year, Konobo district, Liberia, 2012 and 2015

Characteristic	Unweighted^a^			Weighted^b^	
	2012 No. (%) (*n = 364)*	2015 No. (%) (*n = *205)		2012 % (95% CI) (*n* = 364)	2015 % (95% CI) (*n* = 205)
**Maternal age**	30.5 (N/A)^c^	28.6 (N/A)^c^		30.4^c^ (29.6 to 31.1)	28.5^c^ (27.7 to 29.4)
**Maternal education**					
None	99 (27.6)	61 (27.8)		24.8 (20.4 to 29.0)	30.8 (25.5 to 36.7)
Primary	211 (58.8)	117 (57.1)		54.8 (48.2 to 61.2)	57.0 (51.0 to 62.8)
Secondary or higher	49 (13.7)	27 (13.2)		20.4 (14.4 to 28.8)	12.2 (9.0 to 16.4)
**Residents living in a gold mining community**	119 (32.7)	121 (59.0)		58.1 (45.1 to 70.1)	58.2 (49.2 to 66.8)
**Distance to clinic**	26.4 (N/A)^d^	26.1 (N/A)^d^		28.4^d ^(26.6 to 30.2)	26.9^d^ (24.7 to 29.1)
**Community accessible by vehicle**	256 (70.3)	92 (44.9)		47.6 (34.3 to 61.3)	46.5 (37.8 to 55.5)

CI: confidence interval; N/A: not applicable.

^a^ Unweighted counts, means and percentages describe the sample only.

^b^ Weighted values represent the population and are calculated using sampling weights.

^c^ Reported in mean years.

^d^ Reported in mean km.

### Child health

Between 2012 and 2015, the proportion of children receiving health care for childhood illnesses from formal providers significantly increased (Table 2). The adjusted percentage increased by 60.1 points (95% confidence interval, CI: 51.6 to 68.7) for diarrhoea, by 30.6 points (95% CI: 20.5 to 40.7) for fever and by 51.2 points (95% CI: 37.9 to 64.5) for acute respiratory infection. Among those children who received formal provider care in 2015, 83.5% (CI: 74.4 to 89.7) was from a CHW for diarrhoea, 78.6% (95% CI: 64.9 to 87.9) for acute respiratory infection and 80.9% (95% CI: 73.3 to 86.7) for fever. Formal sector care for diarrhoea and fever increased more for children in agricultural than gold-mining communities, but the difference was not statistically significant for acute respiratory infection (Table 3).

**Table 2:** Change in maternal and child health-care use, before and after programme implementation, Konobo district, Liberia, 2012 and 2015

Outcome	Before 2012 Adjusted % (95% CI)^a^	After 2015 Adjusted % (95% CI)^a^	Percentage point difference (95% CI)
**Child health-care use outcomes**			
Diarrhoea treatment from formal provider	6.1 (3.6 to 10.3)	66.3 (56.9 to 74.5)	60.1 (51.6 to 68.7)
Acute respiratory infection treatment from formal provider	6.6 (3.7 to 11.4)	57.8 (44.4 to 70.1)	51.2 (37.9 to 64.5)
Fever treatment from formal provider	26.2 (20.5 to 33.0)	56.8 (49.3 to 64.1)	30.6 (20.5 to 40.7)
**Maternal health-care use outcomes**			
Facility-based delivery	55.8 (49.2 to 62.3)	84.0 (79.1 to 88.0)	28.2 (20.3 to 36.1)
One-or-more antenatal care sessions at a health facility	81.4 (76.6 to 85.4)	82.8 (77.5 to 87.0)	1.4 (−4.7 to 7.5)
Postnatal care (maternal or neonatal) within 24 hours from a clinic staff member or CHW	17.1 (13.0 to 22.1)	19.4 (15.0 to 24.6)	2.3 (−4.2 to 8.8)

CI: confidence interval; CHW: community health worker.

**^a^** Adjusted values were produced using predictive margins after fitting multivariable logistic regression models.

Note**:** Formal care providers included community health workers, ministry of health community health volunteers (who were active in some parts of Liberia but not the pilot programme catchment area) and clinic staff.

**Table 3:** Change in maternal and child health-care use before and after programme implementation, by community type, Konobo district, Liberia, 2012 and 2015

Outcome	Agricultural communities				Mining communities		
	Before 2012 Adjusted % (95% CI)^a^	After 2015 Adjusted % (95% CI)^a^	Percentage point difference (95% CI)		Before 2012 Adjusted % (95% CI)^a^	After 2015 Adjusted % (95% CI)^a^	Percentage point difference (95% CI)
**Child health-care use outcomes**							
Diarrhoea treatment from formal provider	8.5 (4.0 to 17.1)	88.2 (66.9 to 96.5)	79.7 (66.4 to 93.0)		4.4 (1.7 to 11.3)	59.8 (47.0 to 71.4)	55.4 (43.6 to 67.1)
Acute respiratory infection treatment from formal provider	16.3 (5.6 to 39.2)	78.0 (48.7 to 93.0)	61.7 (41.8 to 81.7)		2.0 (0.3 to 10.2)	48.7 (28.3 to 69.6)	46.8 (23.9 to 69.7)
Fever treatment from formal provider	19.4 (12.9 to 28.1)	80.6 (67.4 to 89.3)	61.2 (50.8 to 71.5)		33.1 (23.2 to 44.8)	42.8 (30.6 to 56.0)	9.7 (−4.6 to 23.9)
**Maternal health-care use outcomes**							
Facility-based delivery	48.8 (39.7 to 57.9)	87.8 (79.7.to 92.9)	39.0 (29.8 to 48.2)		61.0 (50.3 to 70.8)	80.6 (72.6 to 86.7)	19.6 (7.6 to 31.5)
One-or-more antenatal care sessions at a health facility	83.4 (77.5 to 88.1)	83.5 (74.3 to 89.9)	0.1 (−7.7 to 7.8)		79.4 (70.6 to 86.0)	82.1 (73.3 to 88.4)	2.7 (−6.6 to 11.9)
Postnatal care (maternal or neonatal) within 24 hours by a clinic staff member or CHW	21.0 (13.9 to 30.6)	25.8 (16.2 to 38.7)	4.8 (−4.2 to 13.9)		14.9 (9.3 to 22.9)	15.5 (9.9 to 23.6)	0.7 (−7.9 to 9.2)

CI: confidence interval; CHW: community health worker.

^a^ Adjusted values were produced using predictive margins after fitting multivariable logistic regression models.

Note: Formal care providers included community health workers, ministry of health community health volunteers (who were active in some parts of Liberia but not the pilot programme catchment area) and clinic staff.

### Maternal health

The adjusted facility-based delivery percentage increased by 28.2 points (95% CI: 20.3 to 36.1; Table 2). The increase was 39.0 percentage points (95% CI: 29.8 to 48.2) in agricultural communities compared to 19.6 points (95% CI: 7.6 to 31.5) in mining communities. There were no significant changes in the receipt of at least one formal provider-associated antenatal care visit or receipt of postnatal care within 24 hours.

## Discussion

Here we evaluate, over a three-year period, the implementation of a programme recruiting and training CHWs to deliver maternal and child care. Despite the Ebola virus disease outbreak, which caused substantial declines in health-care utilization in other regions of the country,^10^^–^^13^ we show increases in health-care use from formal providers for fever, acute respiratory infection and diarrhoea among children and facility-based delivery among pregnant women. Our three-year follow-up period is longer than many prior evaluations.^14^ While many studies do not report distance to clinic, we did not identify any studies from areas as remote as Konobo.^15^

Previous CHW programme evaluations report mixed findings, but are generally positive. CHW programmes improved care seeking for childhood illnesses, though effectiveness and effect sizes vary between interventions.^3^ Similarly, most high-quality impact evaluations report that CHW programmes improve child mortality, but results are mixed and individual studies are often limited because of their relatively small sample size.^14^^,^^16^ Systematic reviews have found substantial heterogeneity in CHW programme components and effects,^3^^,^^14^^,^^16^^,^^17^ suggesting a need for more research on specific programme elements and across contexts.

We did not detect significant improvements in rates of antenatal or postnatal care. Antenatal care rates were already high at baseline (over 80% of pregnancies), which could explain the lack of improvement. The low rates of postnatal care could be explained by a combination of the community focus of the programme and Ebola-related effects on care seeking. However, postnatal care receipt was lower than facility-based deliveries, which suggests missed opportunities at clinics. These results underscore the importance of integrating community and facility-based services throughout the continuum of care.^18^^,^^19^ Additionally, community-based postnatal services may be needed to increase postnatal care rates in remote locations with weak facility-based services.^20^^,^^21^

Programme efficacy was generally lower in gold-mining communities than agricultural communities. In Konobo, mining communities are usually larger and more transient. Limited evidence suggests that CHWs function better with high social capital,^22^^–^^24^ a contextual moderator that is likely reduced in mining communities. Additionally, mining communities tend to have greater availability of private-sector pharmaceutical services. Studies have shown that alternative suppliers replace formal-sector services, particularly when transportation is costly or facility-based services are perceived to be of low quality.^25^^,^^26^

Future investigation will need to assess the sustainability and scalability of these programmes. In addition to sustainable funding, pilot programmes often require alterations to remain appropriate for a wide variety of contexts as they get expanded.^27^^–^^29^ Practices from this programme are being scaled up to over 240 remote communities in adjacent Rivercess County. Furthermore, several of the programme’s features, such as contracts and cash payments, ensuring a CHW-to-population ratio of 1:350, targeting of services to remote communities and field-based supervision, have helped to inform the design of Liberia’s National Community Health Assistant Program.^5^ This programme was launched in 2016 to accelerate progress towards universal health coverage for the most vulnerable populations, especially those in remote communities.^5^ The newly launched programme seeks to transform an existing cadre of unpaid and poorly coordinated CHWs into a more effective workforce by enhancing recruitment, supervision and compensation. The health ministry has organized a coalition of funding and implementation partners to support this new programme. Formal evaluations of both effectiveness and cost–effectiveness are planned as part of the scale-up.

Our study has several limitations. First, results are uncontrolled, limiting causal inferences. However, we are unaware of any other programmes that occurred during implementation and our data show that by 2015 over 75% of child health services were reported to be delivered by CHWs, lending support to a causal inference.^30^ Second, we cannot differentiate the effects of the CHW programme from the effects of the trained traditional midwives’ incentives, transport reimbursements and food stipends, which were simultaneously implemented. Similarly, we cannot identify the independent effects of particular CHW programme sub-elements, such as supervision versus compensation. Third, our programme was done in a single district with a small population, therefore, the results are not generalizable for all remote populations.

This paper offers preliminary data on how an enhanced CHW-based programme was used to promote the uptake of essential maternal and child health services in remote populations. Future investigations will assess the sustainability and scalability of the programme.
